# A New Polymorphism Biomarker rs629367 Associated with Increased Risk and Poor Survival of Gastric Cancer in Chinese by Up-Regulated miRNA-let-7a Expression

**DOI:** 10.1371/journal.pone.0095249

**Published:** 2014-04-23

**Authors:** Qian Xu, Qiguan Dong, Caiyun He, Wenjing Liu, Liping Sun, Jingwei Liu, Chengzhong Xing, Xiaohang Li, Bengang Wang, Yuan Yuan

**Affiliations:** 1 Tumor Etiology and Screening Department of Cancer Institute and General Surgery, the First Affiliated Hospital of China Medical University, and Key Laboratory of Cancer Etiology and Prevention (China Medical University), Liaoning Provincial Education Department, Shenyang, Liaoning, China; 2 Gastrointestinal Surgery and Department 4 of General Surgery, the First Affiliated Hospital of China Medical University, Shenyang, Liaoning, China; 3 Hepatobiliary Surgery and Department 1 of General Surgery, the First Affiliated Hospital of China Medical University, Shenyang, Liaoning, China; Duke Cancer Institute, United States of America

## Abstract

**Background:**

Variant in pri-miRNA could affect miRNA expression and mature process or splicing efficiency, thus altering the hereditary susceptibility and prognosis of cancer. We aimed to assess miRNA-let-7 single nucleotide polymorphisms (SNP) associated with the risk and prognosis of gastric cancer (GC) as predicting biomarkers, and furthermore, its possible mechanisms.

**Methods:**

A two-stage case-control study was designed to screen four miRNA SNPs (pri-let-7a-2 rs629367 and rs1143770, pri-let-7a-1 rs10739971, pri-let-7f-2 rs17276588) in 107 GC patients, 107 atrophic gastritis (AG), and matched 124 controls using PCR-RFLP. Two promising SNPs were validated in another independent 1949 samples (including 579 gastric cancer patients, 649 atrophic gastritis and 721 controls) using Sequenom MassARRAY platform and sequencing.

**Results:**

We found that pri-let-7a-2 rs629367 CC variant genotype was associated with increased risks of gastric cancer and atrophic gastritis by 1.83-fold and 1.86-fold, respectively. For gastric cancer prognosis, patients with rs629367 CC genotype had significantly poorer survival than patients with AA genotype (log-rank *P* = 0.004). We further investigated the let-7a expression levels in serum and found that let-7a expression elevated gradually for rs629367 AA, CA, CC genotype in the atrophic gastritis group (*P* = 0.043). Furthermore, we confirmed these findings in vitro study by overexpressing let-7a carrying pri-let-7a-2 wild-type A or polymorphic-type C allele (*P*<0.001).

**Conclusions:**

pri-let-7a-2 rs629367 CC genotype could increase the risks of gastric cancer as well as atrophic gastritis and was also associated with poor survival of gastric cancer, which possibly by affecting the mature let-7a expression, and could serve as a predicting biomarker for high-risk and poor prognosis of gastric cancer.

## Introduction

MicroRNAs (miRNAs) are 18–25 nucleotide (nt)-long, single-stranded noncoding RNA [1]. Any variation in pri-miRNA, pre-miRNA or mature miRNA may affect the mature process and function of miRNA, which could further affect the expression of hundreds of proteins in the interaction pathway [2]. To some extent, the variant of miRNA played a part as an “oncogene” or “tumor suppressor gene” indirectly [3].

let-7 is the second identified miRNA after the discovery of the first miRNA, lin-4 [4]–[6]. The let-7 family has 10 members including let-7a to let-7g, let-7i, miR-98 and miR-202. The let-7 family plays a crucial role in maintaining the normal physiologic function of human. For example, the pri-miRNA of let-7 family could combine with LIN28 and suppress the splicing procedure of Drosha and Dicer, two important restriction enzymes involving in the mature process for all miRNA [7]. In addition, by knocking down the Drosha enzyme to suppress all the miRNA mature process comprehensively, Kumar at al found that the main reason for the activation and promotion of cell's malignant transformation was the downregulation of let-7 family expression [8]. Although many previous studies have contributed broadly to illustrate the biological functions of let-7 family, few study have focused on genetic variations of members of the family. In fact, if two individuals were selected randomly, their genomes may demonstrate about 0.1% diversity, of which the most common diversity was SNP [9]. Because of the existence of these diversities, the same gene can lead to different gene expression products which could result in different disease susceptibility, hereditary phenotype and prognosis of disease [10].

Previous studies demonstrated that the pri-miRNA SNPs may be used as genetic markers for predicting cancer risk. For example, pri-miR-185 rs2008591 was associated with risk of breast cancer [11]; pri-miR-34b/c rs4938723 was associated with risk of hepatocellular cancer [12]. In the present study, by using data of NCBI bioinformatics databases, we screened all SNPs in the primary precursor area of let-7 family (±600 bp), and found that only 4 SNPs (pri-let-7a-1 rs10739971, pri-let-7a-2 rs629367 and rs1143770, pri-let-7f-2 rs17276588) had Minor Allele Frequency (MAF)>5% in Chinese population, and they were all tagSNPs in Hapmap database. So far, few studies have mentioned the relationship between the four tagSNPs and disease-risk prediction except for schizophrenic illness [13], non-small cell lung cancer [14] and diabetic nephropathy [15]. Their potential role in predicting cancer risk remains largely unknown.

Gastric cancer is the second leading cause of cancer death worldwide and one of the most frequent cancers in East Asian and Chinese populations [16]–[18]. Studies have showed that several miRNA SNPs were associated with gastric cancer risk [19]–[22]. However, whether the above-mentioned four tagSNPs in pri-miRNA genes of let-7 were associated with the risk of gastric cancer and atrophic gastritis in Chinese population, whether it can be used as a predictive genetic biomarker for gastric cancer, and the specific mechanism of how they regulate the disease risk still need to be clarified.

In this study, we first assessed the association between these four candidate tagSNPs in pri-let-7a and susceptibility of gastric cancer and its precursor by conducting a two-stage case-control study in Chinese. Meanwhile, we investigated whether the risk-associated polymorphism contributes toward gastric cancer patients' survival. Furthermore, we examined the effect of the risk-associated polymorphism on regulating its mature miRNA expression in serum and gastric tissue as well as explored its possible regulatory mechanism in modulating disease risk and survival. By conducting the present study, we hope to propose the potential application prospect of the studied SNP as a prewarning biomarker for individuals with high-risk of gastric cancer and its precancerous disease (atrophic gastritis).

## Methods

### Patients and study design

This research project was approved by the Ethical Committee of the First Affiliated Hospital of the China Medical University and the study was divided into three independent but related parts including risk, prognosis and mechanisms research. The risk part was two stages designed, one is screening stage and the other is validated stage. To elucidate the association of candidate SNPs with gastric cancer and atrophic gastritis risks, the screening stage retrospectively recruited samples including 338 cases, consisting of 107 gastric cancer patients, 107 atrophic gastritis cases and 124 matched controls from the First Affiliated Hospital of China Medical University between 2005 and 2010. In the validated stage, we investigated a total of 1949 cases including 579 gastric cancer, 649 atrophic gastritis and 721 healthy controls from a health check program for gastric cancer screening in Zhuanghe of Liaoning Province, China or from the patients in the First Affiliated Hospital of China Medical University, between 2002 and 2013. All the subjects in this study were endoscopically and histologically confirmed. The classification of gastric cancer was divided into intestinal-type and diffuse-type for subgroup analysis which was based on Lauren's classification [23], [24]. The classification and grading of gastritis was based on the Updated Sydney System [25], [26]. Subjects who were endoscopically and histologically confirmed with normal mucosa or only minimal gastritis without other systemic disease or other stomach diseases served as controls. Written informed consents were collected from the patients, and medical histories (including age, sex, smoking, and alcohol consumption) obtained by questionnaire and the records were computerized as previous described [24].

To further investigate the association of risk-associated polymorphism with clinicopathologic parameters and survival in gastric cancer patients, we used data of 150 gastric cancer cases, whose information of death or survival was available. The tumor histological grade was evaluated by World Health Organization criteria and tumors were staged using the 7th edition of the TNM staging system of the International Union Against Cancer (UICC)/American Joint Committee on Cancer (AJCC) (2010) based on postoperative pathologic examination. Patients (i) with distant metastasis found preoperatively, (ii) who underwent preoperative radiotherapy or chemotherapy, or (iii) with incomplete pathological data entries were excluded from the survival analysis. Follow up was completed by August 2013. Finally, 150 patients were included in the survival analysis.

For the evaluation of correlation between risk-associated polymorphism and its miRNA expression in serum, 364 cases including 164 gastric cancer, 100 atrophic gastritis and 100 healthy controls were examined. The characteristics of included subjects were shown in Supplementary Table S1. In addition, for the assessment of correlation between risk-associated polymorphism and its miRNA expression in gastric tissue, 97 non-canceous specimens and 94 gastric cancerous specimens were obtained from 97 patients who underwent gastrectomy at the First Affilicated Hospital of China Medical University between 2009 and 2013.

### Subject's genotyping

Genomic DNA was extracted as described previously with some modifications [27]. The genotyping assay was performed by CapitalBio (Beijing, China) using the Sequenom MassARRAY platform (Sequenom, San Diego, CA, USA) as previously described [24]. 5% of the whole samples were repeatedly genotyped, and the concordance rate was 100%, demonstrating that the genotyping was correct. The detailed materials were shown in the Supplementary Methods.

### The detection of serum H.pylori-IgG titer

According to the method described by the literature [28], serum *H.pylori*-IgG titer was detected by enzyme linked immunosorbent assay (ELISA, Helicobacter pylori IgG kit; Biohit, Helsinki, Finland). The detailed materials were shown in the Supplementary Methods.

### RNA extraction and real-time PCR reaction for miRNA expression in vivo

The miRNA extracted method from the serum and tissue was used as described by the literature [29] with some modifications. The reverse transcription reaction was used One Step Prime Script miRNA cDNA (Perfect Real Time) Kit (TAKARA Biotechnology Co., Ltd, Dalian, China) and Real-time PCR reaction was used miRcute miRNA qPCR detection kit (SYBR) (TIANGEN Biotech Co., Ltd, Beijing, China). The detailed materials were shown in the Supplementary Methods.

### Transient transfection and real-time PCR reaction for miRNA expression in vitro

The commercial expression plasmid pCMV-MIR-let-7a-2 rs629367-C was purchased from Origene Company (Origene Biotech Co., Ltd, Shanghai, China). This plasmid was conducted a site-specific mutagenesis at -216 from C to A (pCMV-let-7a-2 rs629367-A) by Sainuo Company (Sainuo Biotech Co., Ltd, Beijing, China) and confirmed by sequencing. And the candidate cell lines were sequenced to genotype the pri-let-7a-2 rs629367 site to explore whether there was any variant of this rs629367 polymorphism site. Then, the cell lines of the wild-type and also the lowest two let-7a expression, SGC-7901 and AGS, were selected for transfection(More details see Supplementary Methods and Supplementary Figure S2). The human gastric carcinoma cell line, AGS was purchased from ATCC, American Type Culture Collection, USA and SGC-7901 was purchased from the Cell Bank of Chinese Academy of Sciences, Shanghai, China. After 72 hours, the total RNA of cells was extracted and Real-time PCR was used to detected let-7a expression after reverse transcription in order to compare the mature let-7a produced by pCMV-let-7a-2 rs629367-A vs. pCMV-let-7a-2 rs629367-C.

### Statistics

The studied four miRNA polymorphisms were tested for Hardy–Weinberg equilibrium (HWE) among the controls. The continuous variables were shown as mean±standard deviation (SD) and compared by analysis of variance, while the discrete variables were represented as frequencies and percentages and compared by χ2 test [24]. Multivariate logistic regression with adjustments for age, sex and *H. pylori* infection was used to assess the association between miRNA polymorphisms and disease risks. Because smoking and alcohol consumption had nearly a third missing data were not suitable as adjustment factors, only as stratified factors for analysis of the association between the miRNA polymorphisms and disease risks. Univariate and multivariate survival analyses were carried out by the log-rank test and the Cox proportional hazards model. The survival curves were mapped by using the Kaplan–Meier method. Multivariate survival analysis was carried out by adding SNP to all clinicopathological parameters with *P*<0.05. In addition, the copies of miRNA were used lg value for a normal distribution, and the effect of miRNA polymorphisms on its expression levels were tested by the analysis of variance (ANOVA) test. The correlation of let-7a expression in serum and tissue was shown as correlation coefficient by the correlation analysis. Statistical analysis was performed using SPSS version 16.0 software (SPSS, Chicago, IL, USA) and in the screening stage, p values <0.10 was considered significant while all other analyses was considered p values <0.05 as significant.

## Results

### Main effect of miRNA polymorphisms on gastric cancer and atrophic gastritis risk

The genotype frequencies of the studied SNP in the screening stage were shown in Table 1 and the electrophoretogram and sequencing figure of these four miRNA polymorphisms genotypes were shown in Supplementary Figure S1. pri-let-7f-2 rs17276588 was excluded from further analysis because it deviated from HWE. Two SNPs were considered promisingly to be associated with disease risks: the variant genotype frequencies of pri-let-7a-1 rs10739971 between atrophic gastritis and control group showed statistical difference (27.1% vs. 13.7%, *P* = 0.018, Table 1), and the variant genotype frequencies of pri-let-7a-2 rs629367 between gastric cancer and control group also showed difference (1.9% vs.6.5%,*P* = 0.094, Table 1). We considered p value <0.10 as significant and selected this two promising SNP sites into the validated stage.

**Table 1:** The association of four studied pri-miRNA polymorphism and risks of gastric cancer/atrophic gastritis*.

	NCBI Ref(%)	CON(%)	AG(%)	AG vs CON		CON(%)	GC(%)	GC vs CON	
				*P*	OR(95%CI)			*P*	OR(95%CI)
**Screening Stage (Stage 1)**		**n = 124**	**n = 107**			**n = 124**	**n = 107**		
pri-let-7a-1 rs10739971									
GG	12(26.7)	40(32.3)	26(24.3)		1	40(32.3)	32(29.9)		1
GA	26(58.0)	67(54.0)	52(48.6)	0.593	1.19(0.63–2.23)	67(54.0)	59(55.1)	0.819	1.07(0.59–1.93)
AA	7(15.6)	17(13.7)	29(27.1)	**0.018**	**2.59(1.18–5.70)**	17(13.7)	16(15.0)	0.786	1.12(0.49–2.59)
AA VS. GA+GG				**0.015**	**2.34(1.18–4.61)**			0.817	1.06(0.51–2.24)
*P*_HWE_^†^	0.254	0.185	0.778			0.185	0.185		
pri-let-7a-2 rs629367									
AA	62(68.9)	70(56.5)	62(57.9)		1	70(56.5)	64(59.8)		1
CA	26(28.9)	46(37.1)	39(36.4)	0.942	0.98(0.56–1.71)	46(37.1)	41(38.3)	0.948	0.98(0.57–1.69)
CC	2(2.2)	8(6.5)	6(5.6)	0.566	0.71(0.22–2.27)	8(6.5)	2(1.9)	0.106	0.27(0.05–1.32)
CC VS. CA+AA				0.574	0.73(0.24–2.23)			**0.094**	**0.26(0.05–1.26)**
*P*_HWE_^†^	1	0.493	0.337			0.493	0.63		
pri-let-7a-2 rs1143770									
CC	17(40.5)	21(16.9)	26(24.3)		1	21(16.9)	21(19.6)		1
CT	16(38.1)	68(54.8)	52(48.6)	0.285	0.68(0.34–1.38)	68(54.8)	51(47.7)	0.393	0.73(0.36–1.50)
TT	9(21.4)	35(28.2)	51(47.7)	0.672	0.84(0.38–1.88)	35(28.2)	35(32.7)	0.991	1.01(0.46–2.19)
TT VS. CT+CC				0.291	0.70(0.36–1.36)			0.519	0.80(0.41–1.57)
*P*_HWE_^†^	0.2	0.217	0.778			0.217	0.775		
pri-let-7f-2 rs17276588									
GG	60(69.8)	81(65.3)	64(59.8)		1	81(65.3)	70(65.4)		1
GA	14(16.3)	23(18.5)	16(15.0)	0.853	0.93(0.43–2.03)	23(18.5)	18(16.8)	0.974	0.99(0.46–2.12)
AA	12(14.0)	20(16.1)	27(25.2)	0.067	1.38(0.98–1.95)	20(16.1)	19(17.8)	0.846	1.04(0.73–1.48)
*P*_HWE_^†^	0.001	0.000	0.000			0.000	0.000		
**Validation Stage (Stage 2)**									
		**n = 612**	**n = 612**			**n = 501**	**n = 501**		
pri-let-7a2 rs629367									
AA		360(58.8)	364(59.5)		1	303(60.5)	288(57.5)		1
CA		227(37.1)	209(34.2)	0.390	0.89(0.69–1.16)	178(35.5)	181(36.1)	0.690	1.06(0.81–1.39)
CC		25(4.1)	39(6.2)	**0.032**	**1.86(1.06–3.28)**	20(4.0)	32(6.4)	**0.048**	**1.83(1.00–3.32)**
CC Vs. CA+AA				**0.021**	**1.93(1.11–3.36)**			0.057	1.77(0.98–3.18)
pri-let-7a1 rs10739971									
GG		203(33.2)	178(29.1)		1	175(34.9)	167(33.3)		1
GA		300(49.0)	313(51.1)	0.490	1.10(0.83–1.46)	240(47.9)	241(48.1)	0.418	1.13(0.84–1.52)
AA		109(17.8)	121(19.8)	0.364	1.18(0.83–1.68)	86(17.2)	93(18.6)	0.150	1.33(0.90–1.95)
AA Vs. GA+GG				0.700	1.06(0.78–1.46)			0.217	1.24(0.88–1.75)

Note: *using Logistic Regession adjusted by sex, age and *H.pylori* infection status; ^†^means Hardy-Weinberg Equilibrium in population; CON: controls; AG: atrophic gastritis; GC: gastric cancer; NCBI Ref: the reference polymorphism frequencies in Beijing, China in NCBI database.

To confirm the association of pri-let-7a-1 rs10739971 and pri-let-7a-2 rs629367 polymorphisms with gastric cancer and/or atrophic gastritis risks, we re-evaluated these two SNPs in another 1949 cases in the enlarged validated stage. The frequencies of the polymorphisms in all the samples were shown in Supplementary Table S2. Healthy controls were frequency matched to gastric cancer and to atrophic gastritis cases by age (±5 years) and sex (1∶1). After frequency matching, only 501 gastric cancer and 501 controls for gastric cancer risk analysis and 612 atrophic gastritis and 612 controls for atrophic gastritis risk analysis were finally included. In the enlarged validated stage, the variant genotype frequencies of pri-let-7a-2 rs629367 between gastric cancer and control group were statistically different (6.4% vs. 4.0%, Table 1). When compared with the common AA genotype, the variant CC genotype was associated with a 1.83-fold increased risk of gastric cancer (*P* = 0.048, 95%CI = 1.01–3.32,Table 1), and was also associated with a 1.86-fold increased risk of atrophic gastritis (*P* = 0.032, 95%CI = 1.06–3.28, Table 1). In addition, the variant CC genotype was associated with a 1.93-fold increased risk of atrophic gastritis compared with the (AA+AC) genotypes (*P* = 0.021, 95%CI = 1.11–3.36,Table 1), However, we did not observe statistically significant association between pri-let-7a-1 rs10739971 and disease risks in the validated stage.

### Stratified analysis and interation analysis

To further investigate the potential influence of age, sex and environmental factors like status of *H.pylori* infection, smoking and alcohol drinking on genetic effect, we performed stratified analyses for pri-let-7a-1 rs10739971 and pri-let-7a-2 rs629367 polymorphisms based on those factors (Table 2). We observed different effects of pri-let-7a-2 rs629367 on disease risk in different subgroups. Statistical association between CC genotype and increased atrophic gastritis risk were found in females (*P* = 0.025, OR[95%CI] = 2.59[1.13-5.92]), *H. pylori* serology-negative subgroup (*P* = 0.036, OR[95%CI] = 1.97[1.05–3.73]), and in non-drinking subgroup (*P* = 0.023, OR[95%CI] = 2.44[1.13–5.26]). In addition, the CC genotype was found to be associated with increased gastric cancer risk in patients with age≦50 years old (*P* = 0.026, OR[95%CI] = 6.02[1.24–29.10]) and *H. pylori* serology-negative subpopulation (*P* = 0.036, OR[95%CI] = 2.02[1.05–3.90]). However, when we performed the interaction analysis between rs629367 SNP and *H. pylori* infection, smoking and alcohol drinking, no statistical interaction effect was found (*P*_interaction_ = 0.786 and 0.382 respectively, see Supplementary Table S3). No significant association was found between pri-let-7a-1 rs10739971 and gastric cancer or atrophic gastritis risks in any stratified analysis.

**Table 2:** Association of miRNA polymorphisms with risks of gastric cancer/atrophy gastritis stratified by age, sex and environmental factors*.

Variable	Genotype	AG vs CON	*P*	OR(95%CI)^a^	GC vs CON	*P*	OR(95%CI)^a^
		n = 1224			n = 1002		
let-7a-2 rs629367							
Age							
≤50	AA	119/128		1	80/85		1
	CA	76/76	0.872	1.04(0.66–1.63)	37/54	0.303	0.75(0.44–1.29)
	CC	14/4	0.065	3.22(0.93–11.16)	9/2	**0.034**	**5.60(1.14–27.60)**
	CC Vs. CA+AA		0.067	3.15(0.92–10.77)		**0.026**	**6.02(1.24–29.10)**
>50	AA	245/232		1	208/218		1
	CA	133/151	0.232	0.82(0.60–1.13)	144/124	0.261	1.20(0.87–1.64)
	CC	25/21	0.180	1.56(0.81–3.00)	23/18	0.299	1.42(0.73–2.77)
	CC Vs. CA+AA		0.131	1.63(0.86–3.08)		0.365	1.35(0.71–2.59)
Sex							
Male	AA	183/192		1	195/193		1
	CA	130/126	0.605	1.10(0.77–1.56)	116/125	0.411	0.87(0.62–1.21)
	CC	20/15	0.277	1.54(0.71–3.34)	22/15	0.226	1.55(0.76–3.13)
	CC Vs. CA+AA		0.311	1.48(0.69–3.15)		0.174	1.62(0.81–3.23)
Female	AA	181/168		1	93/110		1
	CA	79/101	0.064	0.69(0.47–1.02)	65/53	0.067	1.56(0.97–2.49)
	CC	19/10	0.051	2.32(0.99–5.39)	10/5	0.083	2.74(0.88–8.58)
	CC Vs. CA+AA		**0.025**	**2.59(1.13–5.92)**		0.162	2.22(0.73–6.78)
*H.pylori*							
Negative	AA	135/288		1	164/243		1
	CA	82/173	0.918	1.02(0.73–1.42)	104/141	0.618	1.09(0.79–1.50)
	CC	20/21	**0.041**	**1.97(1.03–3.77)**	23/16	**0.028**	**2.12(1.08–4.14)**
	CC Vs. CA+AA		**0.036**	**1.97(1.05–3.73)**		**0.036**	**2.02(1.05–3.90)**
Positive	AA	229/72		1	124/60		1
	CA	127/54	0.118	0.72(0.47–1.09)	77/37	0.954	0.99(0.60–1.63)
	CC	19/4	0.456	1.53(0.50–4.66)	9/4	0.910	1.07(0.32–3.64)
	CC Vs. CA+AA		0.383	1.63(0.54–4.90)		0.887	1.09(0.33–3.64)
Smoking							
Never Smoker	AA	209/170		1	81/122		1
	CA	114/101	0.419	0.86(0.59–1.25)	53/64	0.163	1.43(0.87–2.35)
	CC	21/13	0.161	1.77(0.80–3.92)	9/8	0.181	2.07(0.71–6.00)
	CC Vs. CA+AA		0.142	1.79(0.82–3.88)		0.266	1.80(0.64–5.07)
Ever Smoker	AA	82/77		1	69/75		1
	CA	54/57	0.750	0.91(0.52–1.59)	50/54	0.966	0.99(0.57–1.72)
	CC	8/6	0.411	1.68(0.49–5.76)	9/6	0.468	1.54(0.48–4.90)
	CC Vs. CA+AA		0.380	1.72(0.51–5.79)		0.355	1.71(0.55–5.33)
Drinking							
Nondrinker	AA	233/192		1	87/145		1
	CA	129/121	0.340	0.84(0.59–1.20)	52/82	0.463	1.19(0.74–1.92)
	CC	25/12	**0.030**	**2.39(1.09–5.27)**	9/7	0.096	2.51(0.85–7.43)
	CC Vs. CA+AA		**0.023**	**2.44(1.13–5.26)**		0.116	2.35(0.81–6.86)
Drinker	AA	58/55		1	41/52		1
	CA	39/37	0.991	1.00(0.54–1.88)	37/36	0.646	1.17(0.60–2.30)
	CC	4/7	0.565	0.67(0.17–2.66)	6/7	0.950	1.04(0.29–3.72)
	CC Vs. CA+AA		0.540	0.65(0.17–2.54)		0.919	1.07(0.31–3.70)
							
let-7a-1 rs10739971							
Age							
≤50	GG	50/66		1	33/48		1
	GA	120/101	0.218	1.39(0.82–2.34)	52/62	0.252	1.43(0.77–2.66)
	AA	39/41	0.486	1.24(0.68–2.27)	33/31	0.086	1.84(0.92–3.71)
	AA Vs. GA+GG		0.842	1.06(0.61–1.83)		0.160	1.53(0.85–2.76)
>50	GG	128/137		1	113/127		1
	GA	193/199	0.976	1.00(0.71–1.39)	175/178	0.777	1.05(0.75–1.48)
	AA	82/68	0.621	1.12(0.72–1.74)	55/55	0.612	1.13(0.71–1.79)
	AA Vs. GA+GG		0.680	1.09(0.74–1.60)		0.672	1.10(0.72–1.67)
Sex							
Male	GG	110/117		1	98/117		1
	GA	162/164	0.632	0.91(0.63–1.33)	152/163	0.581	1.11(0.77–1.59)
	AA	61/52	0.733	1.09(0.66–1.79)	59/53	0.127	1.45(0.89–2.35)
	AA Vs. GA+GG		0.659	1.11(0.71–1.74)		0.149	1.37(0.89–2.10)
Female	GG	68/86		1	48/58		1
	GA	151/136	0.117	1.41(0.92–2.17)	75/77	0.537	1.18(0.70–1.99)
	AA	60/57	0.373	1.26(0.76–2.11)	29/33	0.741	1.12(0.58–2.16)
	AA Vs. GA+GG		0.912	1.03(0.66–1.60)		0.923	1.03(0.57–1.85)
*H.pylori*							
Negative	GG	80/160		1	80/137		1
	GA	112/241	0.686	0.93(0.66–1.32)	116/195	0.902	1.02(0.71–1.47)
	AA	45/81	0.608	1.13(0.71–1.79)	55/68	0.123	1.43(0.91–2.26)
	AA Vs. GA+GG		0.435	1.18(0.78–1.76)		0.095	1.41(0.94–2.10)
Positive	GG	98/43		1	66/38		1
	GA	201/59	0.105	1.47(0.92–2.34)	11/45	0.207	1.41(0.83–2.39)
	AA	76/28	0.484	1.23(0.69–2.16)	33/18	0.791	1.10(0.54–2.25)
	AA Vs. GA+GG		0.810	0.94(0.58–1.54)		0.742	0.90(0.47–1.70)
Smoking							
Never Smoker	GG	100/94		1	42/68		1
	GA	176/149	0.638	1.10(0.74–1.64)	69/101	0.678	1.12(0.66–1.92)
	AA	68/41	0.300	1.33(0.78–2.27)	23/25	0.423	1.36(0.64–2.91)
	AA Vs. GA+GG		0.429	1.21(0.75–1.94)		0.463	1.29(0.65–2.56)
Ever Smoker	GG	42/57		1	35/56		1
	GA	79/63	0.524	1.21(0.67–2.18)	59/60	0.182	1.51(0.83–2.75)
	AA	23/20	0.575	1.25(0.57–2.73)	21/19	0.200	1.70(0.75–3.84)
	AA Vs. GA+GG		0.994	1.00(0.48–2.09)		0.372	1.40(0.67–2.93)
Drinking							
Nondrinker	GG	115/114			47/89		1
	GA	198/167	0.619	1.10(0.75–1.61)	70/118	0.652	1.12(0.68–1.85)
	AA	74/44	0.274	1.33(0.80–2.23)	20/27	0.651	1.19(0.56–2.54)
	AA Vs. GA+GG		0.455	1.19(0.75–1.89)		0.651	1.17(0.59–2.32)
Drinker	GG	27/37		1	17/35		1
	GA	57/45	0.393	1.34(0.68–2.65)	42/43	0.087	1.97(0.91–4.30)
	AA	17/17	0.625	1.24(0.52–2.96)	14/17	0.245	1.84(0.66–5.12)
	AA Vs. GA+GG		0.907	0.95(0.43–2.10)		0.843	1.09(0.46–2.57)

Note: *using Logistic Regession adjusted by the other two factors of sex, age and *H.pylori* infection status; CON: controls; AG: atrophic gastritis; GC: gastric cancer.

### Correlation between pri-let-7a-2 rs629367 polymorphism and clinicopathologic parameters and length of survival of gastric cancer patients

**Figure 1:**
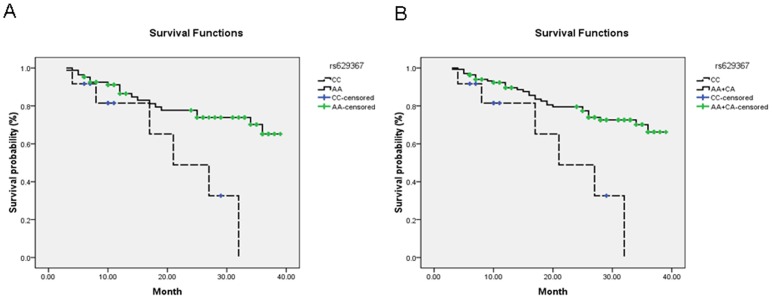
Kaplan–Meier survival curve analysis with the different genotypes of pri-let-7a-2 rs629367. A, CC vs. AA; B, CC vs. AA+CA.

Because of the observation of statistically significant association between rs629367 and gastric cancer and atrophic gastritis risk in the main effect analyses and stratified analyses, we further investigated whether the risk-associated rs629367 correlates with the clinicopathologic phenotype and prognostic survival of gastric cancer. The results showed that no significant correlation of this SNP was found with clinicopathological parameters including tumor size, tumor location, Borrmann type, histologic type, Lauren type, TNM stage, growth pattern, depth of invasion, lymphatic metastasis, *H.pylori* infection status, smoking, drinking, and family history (*P*>0.05, see Supplementary Table S4), but this SNP was found associated with gastric cancer survival. The CC genotype carriers showed unfavorable survival (CC vs. AA: 22.2 months vs. 32.0 months) and increased HRs for gastric cancer patients in univariate analysis (CC vs. AA: *P* = 0.011, HR[95%CI] = 3.39[1.33–8.65]; CC vs. AA+AC: *P* = 0.005, HR[95%CI] = 3.51[1.45–8.49]; Table 3 and Figure 1-A and -B). By adjusting for several clinicopathological parameters which were potential confounding factors correlated with gastric cancer survival (*P*≤0.0001 for tumor size, Borrmann type, TNM stage, depth of invasion and lymphatic metastasis, see Supplementary Table S5), statistically increased HRs were also obtained for CC carriers in the multivariate analysis (CC vs. AA: *P* = 0.004, HR [95%CI] = 4.48[1.60–12.60]; CC vs. AA+AC: *P* = 0.001, HR[95%CI] = 4.69[1.84–11.95], Table 3).

**Table 3:** Multivariate cox proportional hazard analysis for pri-let-7a-2 rs629367 polymorphisms.

	All GC	Death,	MST*	Univariate		Multivariate^‡^	
Factors	n = 150	n = 37	(M)	*P-value*	Hazard ratio (95% CI)	*P-value*	Hazard ratio (95% CI)
rs629367							
AA	83	19	32		1(Ref)		1(Ref)
AC	55	12	31.8^†^	0.697	NA	0.403	NA
CC	12	6	22.2^†^	**0.011**	**3.39(1.33–8.65)**	**0.004**	**4.48(1.60–12.60)**
AC+CC vs. AA				0.652	NA	0.836	NA
CC vs. AA+AC				**0.005**	**3.51(1.45–8.49)**	**0.001**	**4.69(1.84–11.95)**

HR,hazard rate; CI,confidence interval; *, MST, median survival time (months). ^†^, mean survival time was provided when MST could not be calculated. ^‡^, Multivariate survival analysis was carried out by adding the SNP variable to the clinicopathological parameters with *P*<0.05. NA: not available.

### Correlation between rs629367 and mature let-7a expression in serum and gastric tissue

**Figure 2:**
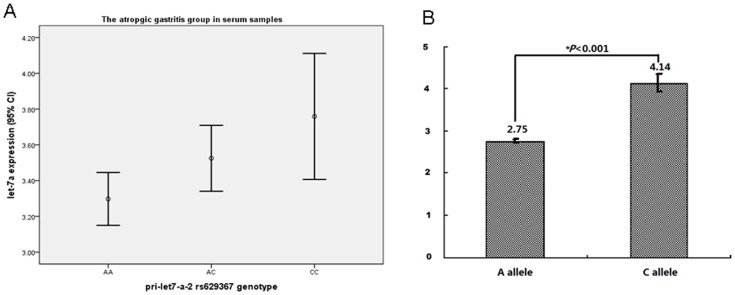
The effect of pri-let-7a rs629367 A/C SNP on the mature let-7a expression in vivo and vitro. A, The let-7a expression of different genotype of pri-let-7a-2 rs629367 in atrophic gastritis group in serum samples. B. the cell mature let-7a expression transinfected by different pri-let-7a-2 rs629367 plasmid. * ***P*** **<0.001**.

In order to study the possible mechanism of rs629367 polymorphism related to gastric cancer, we further investigated the mature let-7a expression in vivo and vitro. The patients' characteristics selected for the study of expression in serum and tissue were shown in Supplementary Table S6. In serum level, stratified by different diseases, we found that in atrophic gastritis group, the mature let-7a expression of rs629367 AA, CA, CC genotype showed a tendency of gradually increasing, and this difference had a statistical significance (3.30±0.56 Vs. 3.52±0.55 Vs. 3.76±0.34, *P* = 0.043, Table 4, Figure 2-A). In tissue level, we analyzed the effect on let-7a SNP genotypes on its mature let-7a expression in gastric cancer tissue, and found rs629367 AA, CA, CC genotype also showed a tendency of gradually increasing, although this difference did not reach statistical significance (Table 4).

**Table 4:** The expression of let-7a and the effect of pri-let-7a-2 rs629367 polymorphism to its mature miRNA expression.

	Serum expression						Tissue expression in situ			
rs629367 Genotype	CON(n)		AG(n)		GC(n)		Cancer Tissue(n)		Noncancer Controls(n)	
	n = 100		n = 100		n = 164		n = 94		n = 97	
AA	54	3.30±0.55	57	3.30±0.56	93	3.40±0.55	62	5.40±0.74	62	5.32±0.72
AC	38	3.46±0.50	37	3.52±0.55	61	3.31±0.51	27	5.40±0.46	27	5.44±0.62
CC	8	3.46±0.59	6	3.76±0.34	10	3.22±0.49	5	5.64±0.62	8	5.64±0.54
P*		0.746		**0.043**		0.427		0.740		0.420

Note: *means the variance analysis was used to calculate the difference between the expressions of three genotypes.

### The mature let-7a expression of pCMV-MIR-pri-let-7a-2 rs629367-A and -C

First, the candidate cell lines were sequenced to genotype the pri-let-7a-2 rs629367 site and found that these candidate cell lines were all rs629367 wild-type (see Supplementary Figure S3). In the vitro level, SGC-7901 and AGS cell lines were transinfected into two plasmids, pCMV-MIR-pri-let-7a-2 rs629367-A and pCMV-MIR-pri-let-7a-2 rs629367-C. After 72 hours, mature let-7a expression had statistical significance in SGC-7901 cell line (*P*<0.001, Figure 2-B). The variant C allele expressed a higher let-7a when compared with the ancient A allele. The AGS cell line got no statistical significance and only showed tendency in accordance with SGC-7901 (see Supplementary Figure S4).

## Discussion

Based on various studies concerning mature miRNAs functions [30], it has been well accepted that miRNA play an important role in the development of cancer as an “oncogene” or “tumor suppressor gene” [31]. However, the relationship between miRNA variants and cancer risk as well as prognosis still needs to be clarified [32]. In the present study, we reported, for the first time, the distribution frequencies of four miRNA tagSNPs (pri-let-7a-1 rs10739971, pri-let-7a-2 rs629367 and rs1143770, pri-let-7f-2 rs17276588) in Northern Chinese. We further validated the promising polymorphism sites in independent and expanded samples and found the variant CC genotype of pri-let-7a-2 rs629367 increased the risks of gastric cancer and atrophic gastritis to 1.83 and 1.86 fold, as well as associated with a poor survival of gastric cancer patients. We further explored the effect of pri-let-7a-2 rs629367 on its expression and the possible mechanisms, and indicated that the mechanism might due to the alternation of the mature let-7a expression, which may eventually alter the susceptibility and prognosis for gastric cancer. To our knowledge, this is the first report about the relationship of rs629367 SNP with susceptibility and prognosis of gastric cancer.

As we know,genetic variations, which arise in miRNA genes including their pri- and pre-miRNA regions, would have opportunity to affect several biologic pathways and influence disease incidence [33]. Thus, pri- and pre-miRNA polymorphisms may be used as genetic markers for predicting cancer risk[32], [34], which have more advantage and strengths than coding gene polymorphisms at the predicting potential because miRNAs frequently located in cancer-associated genomic regions [35] and could regulate almost all the encoding genes [36]. Recently, various studies focused on the pre-miRNA polymorphisms located on the stem-loop, such as pre-mir-196a2 rs11614913 [37], pre-mir-146a rs2910164 [38], pre-mir-499 rs3746444 [39], [40], pre-mir-149 rs2292832 [41], pre-mir-27a rs895819 [42]. Because of the complexity of RNA space structure, increasing attention had been paid to SNP on pri-miRNA. The representative pri-miRNA SNPs were pri-miR-185 rs2008591 associated with breast cancer [11], pri-miR-34b/c rs4938723 associated with hepatocellular cancer [12], pri-miR-30c rs928508 [22] and pri-miR-938 rs2505901 [43] associated with gastric cancer. let-7 families were most previous identified miRNAs [4] and play a crucial role in maintaining the normal physiologic function of human. By using NCBI bioinformatics databases, we found 4 SNPs in the primary precursor area of let-7 family (pri-let-7a-1 rs10739971, pri-let-7a-2 rs629367 and rs1143770, pri-let-7f-2 rs17276588) had Minor Allele Frequency (MAF)>5% in Chinese population, which were all tagSNPs but their potential predicting roles were unclear. In the present study, by screening and validating two stages study we first found that, among 4 tagSNPs, pri-let-7a-2 rs629367 polymorphism was associated with susceptibility of gastric cancer (the variant CC genotype of pri-let-7a-2 rs629367 increased the risks of gastric cancer and atrophic gastritis to 1.83 and 1.86 fold). This pri-let-7a-2 rs629367 SNP was located in downstream 3′-UTR, which was the nearest SNP with the mature miRNA genes among the four screening SNPs, and it would be a new biomarker in predicting gastric cancer risk.

We further performed univariate and multivariate Cox proportional hazards regression analysis of the follow-up data to explore the associations of pri-let-7a-2 rs629367 SNP with overall survival of gastric cancer patients. Consistently significant results were observed from univariate and multivariate Cox models (Table 3). The pri-let-7a-2 rs629367 variant CC genotype carriers showed increased HR with a *P*-value of 0.011 (HR = 3.39) and 0.005 (HR = 3.51) when compared with AA wild-type and AA+AC genotype respectively in univariate analysis. Because several clinicopathological parameters contributed significantly to overall gastric cancer survival (*P*≤0.0001 for tumor size, Borrmann type, TNM stage, depth of invasion and lymphatic metastasis), we performed analysis with adjustment for those potential confounding factors in the multivariate analysis. Eventually, we found the *P*-value of this rs629367 SNP was more significant and statistical HRs were also increased (CC vs. AA: *P* = 0.004, HR = 4.48; CC vs. AA+AC: *P* = 0.001, HR = 4.69, respectively), suggesting that pri-let-7a-2 rs628367 may be an independent risk factor for gastric cancer prognosis.

Several studies revealed that miRNA polymorphisms impair miRNA processing and expression of mature miRNA and play a role in carcinogenesis [12], [33]. In order to study the possible mechanism of rs629367 polymorphism related to gastric cancer, in this study, we investigated the effect of pri-let-7a SNP on the mature let-7a expression both in vivo and vitro. Controlling for the disease factor, we found in the serum atrophic gastritis group, the mature let-7a expression of rs629367 AA, CA, CC genotype showed a significant gradually increase (*P* = 0.043, Table 3). This could partially explain the phenomenon that the variant CC genotype of rs629367 had a higher distribution frequency in atrophic gastritis group than in control group (6.2% vs. 3.9%, Table 1). The relation of rs629367 CC genotype with increased atrophic gastritis risk indicated that this SNP was associated with atrophic gastritis and even gastric cancer possibly by the alternation of the mature miRNA expression, which thereby influences the susceptibility to gastric cancer. On the basis of the expression level in vivo, we further constructed the expressed plasmid containing pri-let-7a-2 rs629367 wild-type A allele and variant C allele in order to observe the mature let-7a expression of the two different allele. We found that after transinfection, SGC-7901 cells containing pCMV-MIR-pri-let-7a-2 rs629367-C allele demonstrated higher mature let-7a expression than that of A allele (*P*<0.001, Figure 1). The result obtained from the vitro experiment was accordant with that of the vivo, which also reveal that pri-let-7a-2 rs629367 polymorphism could affect the mature let-7a expression and alter the susceptibility to gastric cancer. Several studies reported that let-7a was tumor suppressor [44], [45]. We speculated that the variation from A to C might loss its original function of tumor suppressing, which could alter gastric cancer susceptibility and leading to poor survival for prognosis.

Gastric cancer is a multi-factorial disease caused by genetic predisposition as well as environmental factors [46]. To investigate the potential influence of age, sex and environmental factors like status of *H.pylori* infection, smoking and alcohol drinking on genetic effect, we further analyzed the effects of possible influence factors on this pri-let-7a-2 rs629367 SNP, and found pri-let-7a-2 rs629367 was only associated with disease risks in *H. pylori* serology-negative patients group and in non-drinking group. In *H. pylori* serology-negative patients, the ORs for the association of rs629367 SNP and atrophic gastritis and gastric cancer risks elevated to be 2.12 and 1.97 fold, which was higher than the ORs in the whole cases (1.83 and 1.86 fold, respectively). However, there was no statistical significance in *H. pylori* serology-positive patients. Similarly, this phenomenon was also observed in the stratified analysis by drinking: in non-drinking group, the OR for the association of rs629367 SNP and atrophic gastritis risk elevated to be 2.39 fold, while there was no statistical significance in drinking group. This may be because the *H.pylori* and drinking were associated with the incidence of gastric cancer. Brenner et al. had hypothesized that *H.pylori* was a necessary cause for gastric cancer [47], and Tramacere et al. found that there was a strong association between drinking and incidence of gastric cancer [48], [49]. Therefore, the removal of these two environmental factors made the ORs higher than analyzing the whole samples. Furthermore, we performed an interaction analysis of rs629367 SNP with environmental factors including *H. pylori* infection, smoking and drinking but we did not found significant interaction effect with environmental factors such as *H. pylori* infection, smoking and drinking in atrophic gastritis or in gastric cancer (*P*_interaction_>0.05). This phenomenon may be because both pri-let-7a-2 polymorphism and environmental factors contributed to the risk of gastric cancer but the contribution of environmental factors like *H. pylori* infection and drinking was much stronger than the weak effect of gene polymorphisms, and the two factors could not reach an interaction effect of addition or multiplication. And only when removing the environmental factors, the association of polymorphisms with disease risks could demonstrate apparently.

Several limitations still remain in our study: first, in this study, there was a statistical significance of the effect of pri-let-7a-2 rs629367 polymorphism to mature let-7a expression in serum, and we only see the same tendency in tissue but this did not reach statistical difference. The significance of the effect of this rs629367 polymorphism to only serum let-7a expression might be limited and should be further studied and datamined in a larger sample size. Second, the specific functions of this rs629367 required further clarification, for example, whether the variant C has a different biological function from the ancient A; third, the relation of pri-let-7a-2 rs629367 SNP with gastric cancer prognosis observed in this study was exploratory, which requires future larger-scale study to confirm. In the future, larger and multi-centers samples are needed to confirm our findings, and the possible mechanisms need to be clarified by further molecular experiments.

## Conclusions

In summary, our study revealed that pri-let-7a-2 rs629367 CC genotype could increase the risks of gastric cancer and atrophic gastritis and was also associated with poor survival of gastric cancer, which possibly by affecting the mature let-7a expression. The pri-let-7a-2 rs629367 CC genotype might have application prospect to serve as a predicting biomarker for high-risk and poor prognosis of gastric cancer.

## Supporting Information

Figure S1 The electrophoretogram and sequencing figure of four miRNA polymorphisms genotypes. The electrophoretogram and sequencing figure of four miRNA polymorphisms genotypes. A. pri-let-7f-2 rs17276588 SNP: M, 100 bp DNA Marker (TAKARA); Lane 1,3,4,7: GG homozygote; Lane 6: GA heterozygote; Lane 2,5: AA homozygote. A-a, GG homozygote; A-b, GA heterozygote; A-c, AA homozygote. B. pri-let-7a-2 rs1143770 SNP: M, 100 bp DNA Marker (TAKARA); Lane 1,2,6,7: CC homozygote; Lane 3,4,6,7: CT heterozygote. B-a, CC homozygote; B-b, CT heterozygote; B-c, TT homozygote. C. pri-let-7a-1rs10739971 SNP: M, 100 bp DNA Marker (TAKARA); Lane 1,7: GG homozygote; Lane 2,5,6: GA heterozygote; Lane 3,4: AA homozygote. C-a, GG homozygote; C-b, GA heterozygote; C-c, AA homozygote. D. pri-let-7a-2 rs629367 sequencing figure. E-a, AA homozygote; E-b, AC heterozygote; E-c, CC homozygote.
(TIF)

Figure S2 The selection of cell lines for pCMV-MIR-let-7a-A or C plasmid transfection. The selection of cell lines for pCMV-MIR-let-7a-A or C plasmid transfection. This figure showed endogenous let-7a expressed by different cell lines (GES-1, AGS, SGC-7901, BGC-823, BGC-803, MKN-45, N87). The least cell line expressed endogenous let-7a, SGC-7901 and AGS, were selected for transfection because it could reduce the effect that endogenous let-7a participated in the transfection experiments on the other hand could warrant let-7a upstream or downstream pathway molecules existed.
(TIF)

Figure S3 The pri-let-7a-2 rs629367 genotype of the candidate cell lines. The pri-let-7a-2 rs629367 genotype of the candidate cell lines was sequenced. The human gastric cell lines, GES-1, AGS, SGC-7901, BGC-803 and NCI-N87 was all rs629367 A allele, which suggested no variant genotype among them for interference.
(TIF)

Figure S4 The mature let-7a expression in AGS cell line transinfected by different pri-let-7a-2 rs629367 plasmid.
(TIF)

Table S1 The demographic geography characteristics.
(DOC)

Table S2 The frequencies of pri-let-7a-2 rs629367 and pri-let-7a-1 rs10739971 polymorphism in the MassArray assay.
(DOC)

Table S3 The interaction of pri-let-7a-2 rs629367 polymorphism and environmental factors in risks of gastric cancer/atrophic gastritis.
(DOC)

Table S4 Associations between genotype distributions of pri-let-7a-1 rs107399 and clinicopathological parameters of gastric cancer (n = 150).
(DOC)

Table S5 Gastric cancer patient clinical features and univariate analysis of overall survival.
(DOC)

Table S6 The patients' characteristics selected for the study of expression in serum and tissue.
(DOC)

Methods S1 (DOC)
