# Supplementary figures and images for: A New Polymorphism Biomarker rs629367 Associated with Increased Risk and Poor Survival of Gastric Cancer in Chinese by Up-Regulated miRNA-let-7a Expression
Source: PLoS One. 2014 Apr 23;9(4):e95249. doi: 10.1371/journal.pone.0095249 (PMC3997364; doi:10.1371/journal.pone.0095249)

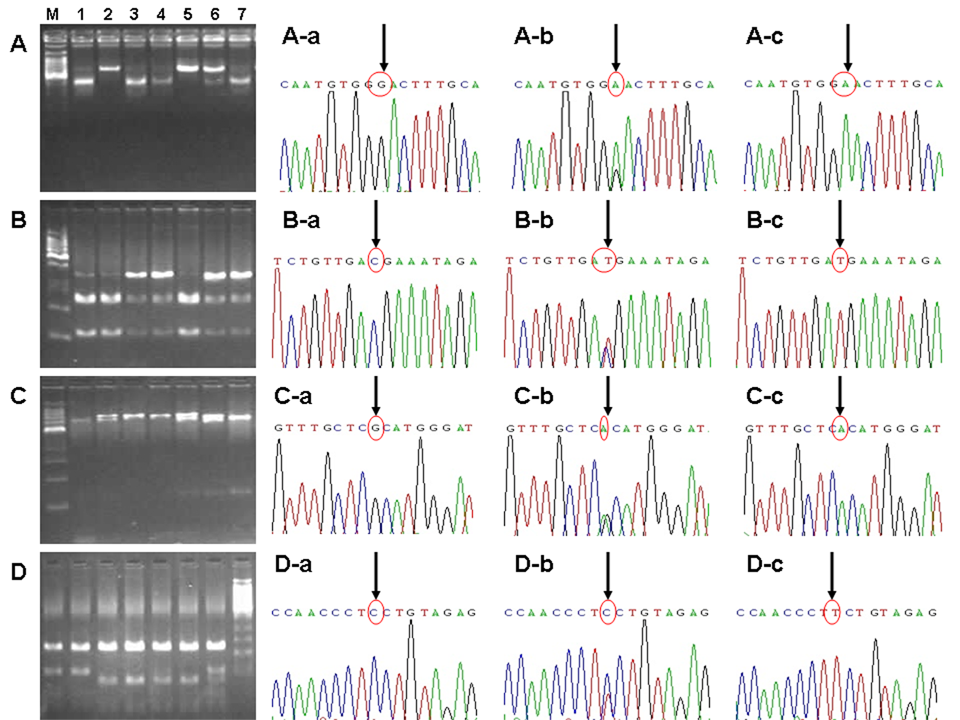

Supplement: Figure S1 — The electrophoretogram and sequencing figure of four miRNA polymorphisms genotypes. The electrophoretogram and sequencing figure of four miRNA polymorphisms genotypes. A. pri-let-7f-2 rs17276588 SNP: M, 100 bp DNA Marker (TAKARA); Lane 1,3,4,7: GG homozygote; Lane 6: GA heterozygote; Lane 2,5: AA homozygote. A-a, GG homozygote; A-b, GA heterozygote; A-c, AA homozygote. B. pri-let-7a-2 rs1143770 SNP: M, 100 bp DNA Marker (TAKARA); Lane 1,2,6,7: CC homozygote; Lane 3,4,6,7: CT heterozygote. B-a, CC homozygote; B-b, CT heterozygote; B-c, TT homozygote. C. pri-let-7a-1rs10739971 SNP: M, 100 bp DNA Marker (TAKARA); Lane 1,7: GG homozygote; Lane 2,5,6: GA heterozygote; Lane 3,4: AA homozygote. C-a, GG homozygote; C-b, GA heterozygote; C-c, AA homozygote. D. pri-let-7a-2 rs629367 sequencing figure. E-a, AA homozygote; E-b, AC heterozygote; E-c, CC homozygote. (TIF) [file pone.0095249.s001.tif]

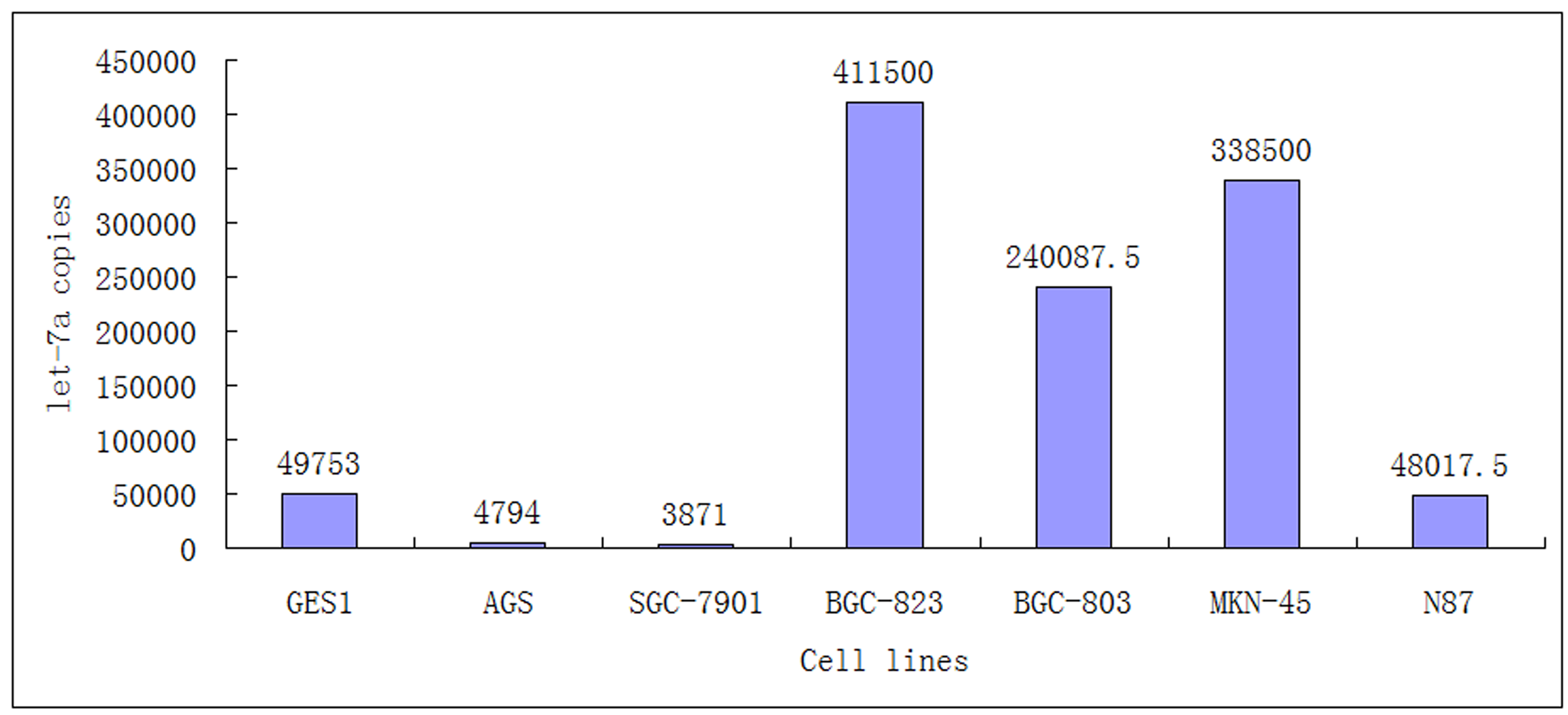

Supplement: Figure S2 — The selection of cell lines for pCMV-MIR-let-7a-A or C plasmid transfection. The selection of cell lines for pCMV-MIR-let-7a-A or C plasmid transfection. This figure showed endogenous let-7a expressed by different cell lines (GES-1, AGS, SGC-7901, BGC-823, BGC-803, MKN-45, N87). The least cell line expressed endogenous let-7a, SGC-7901 and AGS, were selected for transfection because it could reduce the effect that endogenous let-7a participated in the transfection experiments on the other hand could warrant let-7a upstream or downstream pathway molecules existed. (TIF) [file pone.0095249.s002.tif]

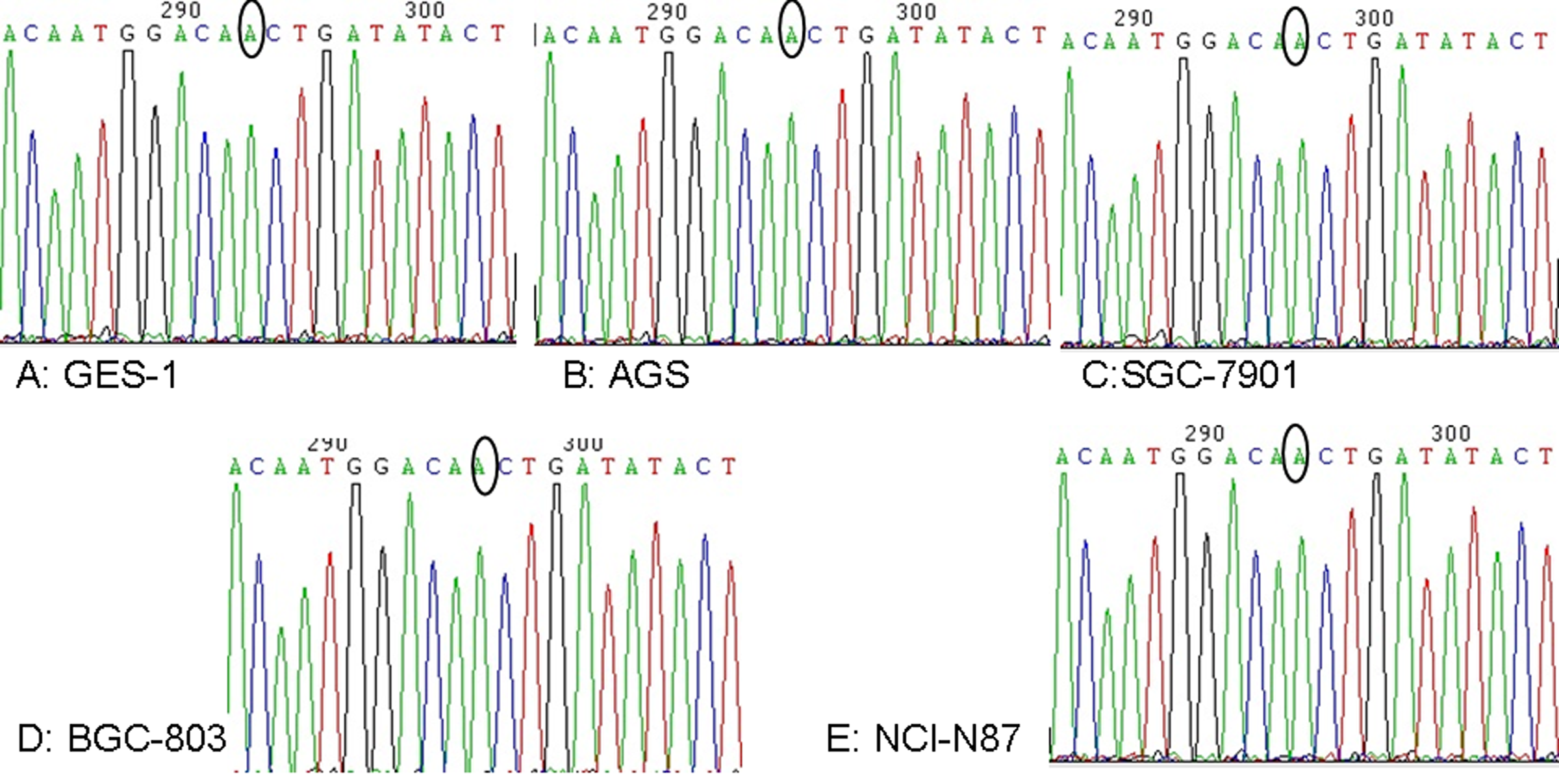

Supplement: Figure S3 — The pri-let-7a-2 rs629367 genotype of the candidate cell lines. The pri-let-7a-2 rs629367 genotype of the candidate cell lines was sequenced. The human gastric cell lines, GES-1, AGS, SGC-7901, BGC-803 and NCI-N87 was all rs629367 A allele, which suggested no variant genotype among them for interference. (TIF) [file pone.0095249.s003.tif]

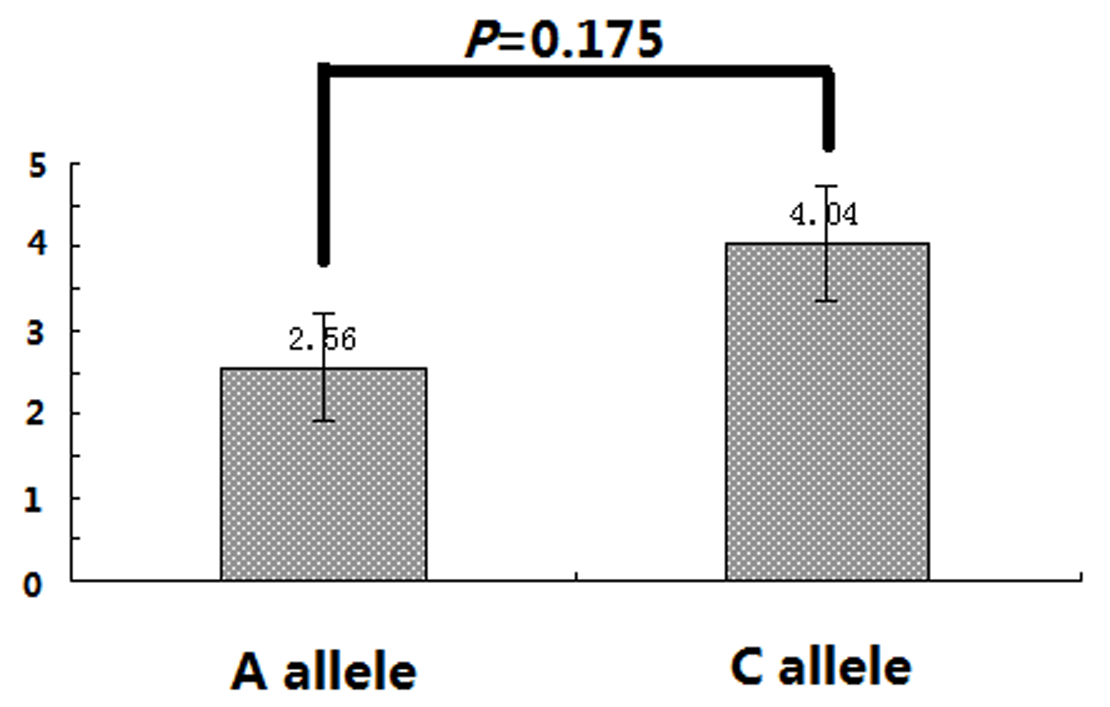

Supplement: Figure S4 — The mature let-7a expression in AGS cell line transinfected by different pri-let-7a-2 rs629367 plasmid. (TIF) [file pone.0095249.s004.tif]
