# Supplementary material for: A New Polymorphism Biomarker rs629367 Associated with Increased Risk and Poor Survival of Gastric Cancer in Chinese by Up-Regulated miRNA-let-7a Expression
Source: PLoS One. 2014 Apr 23;9(4):e95249. doi: 10.1371/journal.pone.0095249 (PMC3997364; doi:10.1371/journal.pone.0095249)
Supplement: Table S1 — The demographic geography characteristics. (DOC) [file pone.0095249.s005.doc]

**Supplementary Table S1**: The demographic geography characteristics

| Variables | AG vs. CON | |  | GC vs. CON | |
| --- | --- | --- | --- | --- | --- |
|  | CON(%) | AG(%) |  | CON(%) | GC(%) |
| **Screening set(Stage 1)** | | |  |  |  |
|  | **n=124** | **n=107** |  | **n=124** | **n=107** |
| Gender | P=0.803 |  |  | P=0.964 |  |
| Male | 78(62.9) | 69(64.5) |  | 78(62.9) | 67(62.6) |
| Female | 46(37.1) | 38(35.5) |  | 46(37.1) | 40(37.4) |
| Age | P=0.029 |  |  | P=0.029 |  |
| Mean±SD | 58.42±11.67 | 60.71±11.24 |  | 58.42±11.67 | 60.42±11.05 |
| Median | 55 | 56 |  | 55 | 57 |
| Range | 23-81 | 28-83 |  | 23-81 | 27-80 |
| H.pylori | P=0.010 |  |  | P=0.190 |  |
| positive | 44(35.5) | 56(52.3) |  | 44(35.5) | 47(43.9) |
| negetive | 80(64.5) | 51(47.7) |  | 80(64.5) | 60(56.1) |
|  |  |  |  |  |  |
| **Validation set(Stage 2)** | | |  |  |  |
| **Total** | **n=721** | **n=649** |  | **n=721** | **n=579** |
| Sex | *P*=0.032 | |  | *P*＜0.001 | |
| Male | 366(50.8) | 367(56.5) |  | 366(50.8) | 388(67.0) |
| Female | 355(49.2) | 282(43.5) |  | 355(49.2) | 191(33.0) |
| Age | *P*＜0.001 | |  | *P*＜0.001 | |
| Mean±SD | 53.07±9.85 | 55.04±8.96 |  | 53.07±9.85 | 59.06±11.13 |
| Age Range | 17-85 | 16-82 |  | 17-85 | 26-87 |
| *H.pylori* | *P*＜0.001 | |  | *P*＜0.001 | |
| Positive | 153(21.2) | 398(61.3) |  | 153(21.2) | 246(42.5) |
| Negative | 568(78.8) | 251(38.7) |  | 568(78.8) | 333(57.5) |
| Smoking | **n=497** | **n=520** |  | **n=497** | **n=315** |
|  | *P*=0.773 | |  | *P*<0.001 | |
| Ever Smoker | 145(29.2) | 156(30.0) |  | 145(29.2) | 145(46.0) |
| Never Smoker | 352(70.8) | 364(70.0) |  | 352(70.8) | 170(54.0) |
| Drinking | **n=497** | **n=520** |  | **n=497** | **n=274** |
|  | *P*=0.528 | |  | *P*<0.001 | |
| Drinker | 100(20.1) | 113(21.7) |  | 100(20.1) | 102(37.2) |
| Nondrinker | 397(79.9) | 407(78.3) |  | 397(79.9) | 172(62.8) |
|  |  |  |  |  |  |
| **Matched Cases** | **n=612** | **n=612** |  | **n=501** | **n=501** |
| Sex | *P*=1.000 | |  | *P*=1.000 | |
| Male | 333(54.4) | 333(54.4) |  | 333(66.5) | 333(66.5) |
| Female | 279(45.6) | 279(45.6) |  | 168(33.5) | 168(33.5) |
| Age | *P*=0.581 | |  | *P*=0.777 | |
| Mean±SD | 54.55±9.02 | 54.84±9.00 |  | 56.33±8.65 | 56.50±8.79 |
| Age Range | 17-85 | 16-82 |  | 29-85 | 30-84 |
| *H.pylori* | *P*＜0.001 | |  | *P*＜0.001 | |
| Positive | 130(21.2) | 375(61.3) |  | 101(20.2) | 210(41.9) |
| Negative | 482(78.8) | 237(38.7) |  | 400(79.8) | 291(58.1) |
|  |  |  |  |  |  |
| Smoking | **n=424** | **n=488** |  | **n=329** | **n=271** |
|  | *P*=0.015 | |  | *P*=0.128 | |
| Ever Smoker | 140(33.0) | 144(29.5) |  | 135(41.0) | 128(47.2） |
| Never Smoker | 284(67.0) | 344(70.5) |  | 194(59.0) | 143(52.8) |
|  |  |  |  |  |  |
| Drinking | **n=329** | **n=232** |  | **n=329** | **n=232** |
|  | *P*=0.004 | |  | *P*=0.067 | |
| Drinker | 99(23.3) | 101(20.7) |  | 95(28.9) | 84(36.2) |
| Nondrinker | 325(76.7) | 387(79.3) |  | 234(71.1) | 148(63.8) |

CON: controls; AG: atrophic gastritis; GC: gastric cancer
