# Supplementary material for: A New Polymorphism Biomarker rs629367 Associated with Increased Risk and Poor Survival of Gastric Cancer in Chinese by Up-Regulated miRNA-let-7a Expression
Source: PLoS One. 2014 Apr 23;9(4):e95249. doi: 10.1371/journal.pone.0095249 (PMC3997364; doi:10.1371/journal.pone.0095249)
Supplement: Table S2 — The frequencies of pri-let-7a-2 rs629367 and pri-let-7a-1 rs10739971 polymorphism in the MassArray assay. (DOC) [file pone.0095249.s006.doc]

**Supplementary Table** S**2**: the frequencies of pri-let-7a-2 rs629367 and pri-let-7a-1 rs10739971 polymorphism detected in the MassArray assay

|  | CON(%) | AG(%) | GC(%) |
| --- | --- | --- | --- |
|  | **n=721** | **n=649** | **n=579** |
| pri-let-7a2 rs629367 |  |  |  |
| AA | 421(58.4) | 386(59.5) | 330(57.0) |
| CA | 271(37.6) | 221(34.1) | 211(36.4) |
| CC | 29(4.0) | 42(6.5) | 38(6.6) |
| pri-let-7a1 rs10739971 |  |  |  |
| GG | 239(33.1) | 191(29.4) | 194(33.5) |
| GA | 353(49.0) | 330(50.8) | 279(48.2) |
| AA | 129(17.9) | 128(19.7) | 106(18.3) |
