# Supplementary material for: A New Polymorphism Biomarker rs629367 Associated with Increased Risk and Poor Survival of Gastric Cancer in Chinese by Up-Regulated miRNA-let-7a Expression
Source: PLoS One. 2014 Apr 23;9(4):e95249. doi: 10.1371/journal.pone.0095249 (PMC3997364; doi:10.1371/journal.pone.0095249)
Supplement: Table S3 — The interaction of pri-let-7a-2 rs629367 polymorphism and environmental factors in risks of gastric cancer/atrophic gastritis. (DOC) [file pone.0095249.s007.doc]

**Supplementary Table S3:** The interaction of pri-let-7a-2 rs629367polymorphism and environmental factors in risks of gastric cancer/atrophic gastritis *

|  |  |  | AG vs CON(n=1224) | | |  | GC vs CON(n=1002) | |
| --- | --- | --- | --- | --- | --- | --- | --- | --- |
|  |  |  | *P* | | OR(95%CI) ^a^ |  | *P* | OR(95%CI) ^a^ |
| *H.pylori* | (-) | AA+CA |  | | 1 |  |  | 1 |
|  |  | CC | 0.029 | | 2.02(1.07-3.81) |  | 0.031 | 2.06(1.07-3.97) |
|  | (+) | AA+CA | <0.001 | | 6.00(4.63-7.78) |  | <0.001 | 2.97(2.23-3.96) |
|  |  | CC | <0.001 | | 10.09(3.39-30.02) |  | 0.053 | 3.22(0.98-10.58) |
|  |  | *P*_interaction_ | 0.786 | |  |  | 0.382 |  |
| Smoking | Never Smoker | AA+CA |  | | 1 |  |  | 1 |
|  |  | CC | 0.373 | | 1.35(0.70-2.62) |  | 0.354 | 1.45(0.66-3.20) |
|  | Ever Smoker | AA+CA | 0.824 | | 1.03(0.78-1.36) |  | <0.001 | 2.04(1.50-2.76) |
|  |  | CC | 0.345 | | 1.64(0.59-4.56) |  | 0.009 | 3.87(1.41-10.66) |
|  |  | *P*_interaction_ | | 0.983 |  |  | 0.913 |  |
| Drinking | Nondrinker | AA+CA |  | | 1 |  |  | 1 |
|  |  | CC | 0.102 | | 1.80(0.89-3.65) |  | 0.150 | 2.10(0.77-5.77) |
|  | Drinker | AA+CA | 0.574 | | 0.91(0.66-1.26) |  | 0.051 | 1.45(0.99-2.10) |
|  |  | CC | 0.264 | | 0.49(0.14-1.70) |  | 0.553 | 1.40(0.46-4.25) |
|  |  | *P*_interaction_ | 0.122 | |  |  | 0.341 |  |

Note: **P* value was using Logistic Regession adjusted by sex and age. CON: controls; AG: atrophic gastritis; GC: gastric cancer
