# Supplementary material for: A New Polymorphism Biomarker rs629367 Associated with Increased Risk and Poor Survival of Gastric Cancer in Chinese by Up-Regulated miRNA-let-7a Expression
Source: PLoS One. 2014 Apr 23;9(4):e95249. doi: 10.1371/journal.pone.0095249 (PMC3997364; doi:10.1371/journal.pone.0095249)
Supplement: Table S4 — Associations between genotype distributions of pri-let-7a-1 rs107399 and clinicopathological parameters of gastric cancer (n = 150). (DOC) [file pone.0095249.s008.doc]

**Supplementary Table S4** Associations between genotype distributions of pri-let-7a-2 rs629367 and clinicopathological parameters of gastric cancer (n=150)

| Parameters | AA+AC | CC | *P-value* |
| --- | --- | --- | --- |
| Age |  |  | 0.480 |
| Sex |  |  |  |
| Male | 91(65.9) | 9(75.0) |  |
| Female | 47(34.1) | 3(25.0) | 0.523 |
| Size |  |  |  |
| ≤4cm | 66(47.8) | 6(50.0) |  |
| ＞4cm | 72(52.2) | 6(50.0) | 0.885 |
| Location |  |  |  |
| Upper | 26(18.8) | 3(25.0) |  |
| Middle | 28(20.3) | 2(16.7) |  |
| Lower | 79(57.2) | 7(58.3) |  |
| Entire | 5(3.6) | 0(0.0) | 0.867 |
| Macroscopic type |  |  |  |
| Early stage | 25(18.1) | 2(16.7) |  |
| Borrmann Ⅰ | 3(2.0) | 0(0.0) |  |
| Borrmann Ⅱ | 13(8.7) | 2(16.7) |  |
| Borrmann Ⅲ | 90(60.0) | 6(50.0) |  |
| Borrmann Ⅳ | 17(11.3) | 2(16.7) | 0.776 |
| Differentiation |  |  |  |
| Well/moderate | 49(42.2) | 5(55.6) |  |
| Poor | 67(57.8) | 4(44.4) | 0.437 |
| Lauren grade |  |  |  |
| Intestinal | 48(35.0) | 5(45.5) |  |
| Diffuse | 89(65.0) | 6(54.5) | 0.488 |
| Unclassified | 1 | 1 |  |
| TNM stage |  |  |  |
| Ⅰ | 34(24.6) | 4(33.3) |  |
| Ⅱ | 17(12.3) | 0(0.0) |  |
| Ⅲ | 27(19.6) | 3(25.0) |  |
| Ⅳ | 60(43.5) | 5(41.7) | 0.577 |
| Growth pattern |  |  |  |
| Massive | 16(11.6) | 0(0.0) |  |
| Nested | 49(35.5) | 3(25.0) |  |
| Diffused | 73(52.9) | 9(75.0) | 0.255 |
| Depth of invasion |  |  |  |
| Mucous and submucosal layer | 26(18.8) | 4(33.3) |  |
| Muscular and subserosa layer | 28(20.3) | 2(16.7) |  |
| Serosal layer | 76(55.1) | 5(41.7) |  |
| Serosa invasion adjacent organs | 8(5.8) | 1(8.3) | 0.633 |
| Lymphatic metastasis |  |  |  |
| Positive | 85(61.6) | 7(58.3) |  |
| Negative | 53(38.4) | 5(41.7) | 0.824 |
| Smoking |  |  |  |
| Ever Smoker | 51(37.0) | 6(50.0) |  |
| Never Smoker | 87(63.0) | 6(50.0) | 0.372 |
| Drinking |  |  |  |
| Drinker | 44(31.9) | 4(33.3) |  |
| Nondrinker | 94(68.1) | 8(66.7) | 0.918 |
| Family history |  |  |  |
| Yes | 26(18.8) | 1(8.3) |  |
| No | 112(81.2) | 11(91.7) | 0.363 |
| *H. pylor*i-IgG |  |  |  |
| Positive | 84(60.9) | 8(66.7) |  |
| Negative | 54(39.1) | 4(33.3) | 0.692 |
