# Supplementary material for: A New Polymorphism Biomarker rs629367 Associated with Increased Risk and Poor Survival of Gastric Cancer in Chinese by Up-Regulated miRNA-let-7a Expression
Source: PLoS One. 2014 Apr 23;9(4):e95249. doi: 10.1371/journal.pone.0095249 (PMC3997364; doi:10.1371/journal.pone.0095249)
Supplement: Table S5 — Gastric cancer patient clinical features and univariate analysis of overall survival. (DOC) [file pone.0095249.s009.doc]

**Supplementary Table S5**: Gastric cancer patient clinical features and univariate analysis of overall survival

|  | All GC | Death, | MST* |  |
| --- | --- | --- | --- | --- |
| Factors | (%), n=150 | n=37 | (M) | *P-value* |
| Age |  |  |  | 0.548 |
| Sex |  |  |  |  |
| Male | 100(66.7) | 23 | 32.8† |  |
| Female | 50(33.3) | 14 | 24.7† | 0.158 |
| Size |  |  |  |  |
| ≤4cm | 72(48.0) | 5 | 36.8† |  |
| ＞4cm | 78(52.0) | 32 | 32.0 | **6×10^-7^** |
| Location |  |  |  |  |
| Upper | 29(19.3) | 7 | 29.0† |  |
| Middle | 30(20.0) | 4 | 32.0† |  |
| Lower | 86(57.3) | 22 | 32.1† |  |
| Entire | 5(3.3) | 4 | 26.0 | 0.051 |
| Macroscopic type |  |  |  |  |
| Early stage | 27(18.0) | 0 |  |  |
| Borrmann Ⅰ | 3(2.0) | 0 |  |  |
| Borrmann Ⅱ | 13(8.7) | 2 | 33.8† |  |
| Borrmann Ⅲ | 90(60.0) | 23 | 31.2† |  |
| Borrmann Ⅳ | 17(11.3) | 12 | 17.0 | **2×10^-7^** |
| Differentiation |  |  |  |  |
| Well/moderate | 54(36.0) | 13 | 33.6† |  |
| Poor | 71(47.3) | 18 | 29.5† | 0.295 |
| Lauren grade |  |  |  |  |
| Intestinal | 53(35.3) | 13 | 30.0† |  |
| Diffuse | 95(63.3) | 22 | 33.4† | 0.444 |
| Unclassified | 2(1.3) | 2 |  |  |
| TNM stage |  |  |  |  |
| Ⅰ | 38(25.3) | 0 |  |  |
| Ⅱ | 17(11.3) | 0 |  |  |
| Ⅲ | 30(20.0) | 5 | 25.0 |  |
| Ⅳ | 65(43.3) | 32 | 32.6† | **2×10^-10^** |
| Growth pattern |  |  |  |  |
| Massive | 16(10.7) | 1 | 36.6† |  |
| Nested | 52(34.7) | 16 | 31.7† |  |
| Diffused | 82(54.6) | 20 | 29.1† | 0.108 |
| Depth of invasion |  |  |  |  |
| Mucous and submucosal layer | 30(20.0) | 0 |  |  |
| Muscular and subserosa layer | 30(20.0) | 3 | 36.4† |  |
| Serosal layer | 81(54.0) | 29 | 34.0 |  |
| Serosa invasion adjacent organs | 9(6.0) | 5 | 21.0 | **6×10^-7^** |
| Lymphatic metastasis |  |  |  |  |
| Positive | 92(61.3) | 33 | 34.0 |  |
| Negative | 58(38.7) | 4 | 36.8† | **1×10^-4^** |
| Smoking |  |  |  |  |
| Ever Smoker | 57(38.0) | 11 | 32.6† |  |
| Never Smoker | 93(62.0) | 26 | 30.8† | 0.313 |
| Drinking |  |  |  |  |
| Drinker | 48(32.0) | 11 | 36.0 |  |
| Nondrinker | 102(68.0) | 26 | 31.1† | 0.544 |
| Family history |  |  |  |  |
| Yes | 27(18.0) | 4 | 33.9† |  |
| No | 123(82.0) | 33 | 30.9† | 0.110 |
| *H. pylor*i-IgG |  |  |  |  |
| Positive | 92(61.3) | 28 | 31.3† |  |
| Negative | 58(38.7) | 9 | 32.6† | 0.294 |
| *, MST, median survival time (months). †, mean survival time was provided when MST could not be calculated. | | | | |
