# Supplementary material for: A New Polymorphism Biomarker rs629367 Associated with Increased Risk and Poor Survival of Gastric Cancer in Chinese by Up-Regulated miRNA-let-7a Expression
Source: PLoS One. 2014 Apr 23;9(4):e95249. doi: 10.1371/journal.pone.0095249 (PMC3997364; doi:10.1371/journal.pone.0095249)
Supplement: Table S6 — The patients' characteristics selected for the study of expression in serum and tissue. (DOC) [file pone.0095249.s010.doc]

**Supplementary Table S6: The patients’ characteristics selected for the study of expression in serum and tissue**

| Variables | Serum | | |  | Tissue | |
| --- | --- | --- | --- | --- | --- | --- |
|  | CON(%) | AG(%) | GC(%) |  | Cancer Tissue(%) | Noncancer Controls(%) |
|  | n=100 | n=100 | n=164 |  | n=94 | n=97 |
| Sex | *P*=0.993 | | |  |  |  |
| Male | 64(64.0) | 64(64.0) | 104(63.4) |  | 63(67.0) | 66(68.0) |
| Female | 36(36.0) | 36(36.0) | 60(36.6) |  | 31(33.0) | 31(32.0) |
| Age | *P=*0.877 | | |  |  |  |
| Mean±SD | 60.0±11.2 | 60.6±11.1 | 60.0±11.0 |  | 59.3±11.5 | 59.4±11.1 |
| Age Range | 23-81 | 28-83 | 27-80 |  | 30-87 | 30-87 |
| *H.pylori* | *P=*0.038 | | |  |  |  |
| Positive | 64(64.0) | 54(54.0) | 74(45.4) |  | 46(48.9) | 47(48.5) |
| Negative | 36(36.0) | 46(46.0) | 89(54.6) |  | 45(47.9) | 46(47.4) |
| Missing |  |  |  |  | 3(3.2) | 4(4.1) |
