# Supplementary material for: A New Polymorphism Biomarker rs629367 Associated with Increased Risk and Poor Survival of Gastric Cancer in Chinese by Up-Regulated miRNA-let-7a Expression
Source: PLoS One. 2014 Apr 23;9(4):e95249. doi: 10.1371/journal.pone.0095249 (PMC3997364; doi:10.1371/journal.pone.0095249)
Supplement: Methods S1 — (DOC) [file pone.0095249.s011.doc]

**Supplementary Methods**

*Subject’s genotyping*

1.DNA extracted(Xu et al., 2009)

Genomic DNA was extracted from frozen clot (500µL), and added 800µL of TE buffer (triethanolamide), mixed well, centrifuged at 10000×g for 5 minutes to disperse the clots. Following clot disruption, 400µL of TE, 25 µL of 10% SDS and 5µL of 20mg/mL proteinase K were put into the residual clot material and incubated at 37℃ overnight. The supernatant was extracted and an equal volume of phenol was added. The tube was placed on a rotator for 15 minutes to extract DNA. After rotation, the tube was centrifuged at 10,000×g for 15 mins. The supernatant was poured off and the second extraction was performed with the addition of an equal volume of a mixture of phenol and chloroform (1:1) to the tube. Following centrifugation, the supernatant was poured off and the third extraction was performed with the addition of an equal volume of chloroform. Following centrifugation, the supernatant was absorbed and two volumes of protein precipitation solution (two volumes of absolute ethanol containing 10% 3mol/L sodium acetate) were added and incubated for 2 hr at -20 °C. Each sample was centrifuged at 10,000×g for 10 mins. After centrifugation, the resulting DNA pellet was rinsed with 75% ethanol and centrifuged at 10,000×g for 10 mins. The 75% ethanol was decanted and the tube inverted on clean absorbent paper for 30mins. The resulting DNA was reconstituted in a TE buffer and stored at -20° until use.

2. PCR-RFLP (Polymerase Chain Reaction-Restriction Fragment Length Polymorphism)

The Primers was designed by the software Primer 5.0 and the restriction enzyme was found by the software Watcut (<http://watcut.uwaterloo.ca/watcut/watcut/template.php>). The total PCR reaction volume was 25 μL containing 2.5 μL of 10×PCR buffer, 2 μL of 2.5 mM dNTP mixture, 1 μL of each upstream and downstream primer at 10 pmol/μL, 2.5 U of rTaq DNA polymerase, 10 ng of the template DNA, and an appropriate amount of ddH_2_O. PCR reaction was performed by pre-denaturing at 95 °C for 1 min, 35 cycles with 95 °C for 30 s, 58-61 °C for 30 s, 72 °C for 30 s (See in Supplementary Table 5). An amount of 5 μL of PCR product was incubated overnight at 37℃ with restriction endonuclease (See in Supplementary Table 5). All these cleavaged products mixed with GeneFinder (Bio-V Inc., Xiamen, China) underwent electrophoresis in 2.0% agarose gel at 150 V for 45 min separation and observed. PCR products on the three different genotypes of each miRNA were commercially sequenced using an ABI377 DNA Sequencer (ABI, USA). The other reagents were purchased from TaKaRa Inc., Dalian, China.

| No. | miRNA | primer name | primer sequence | Restriction enzyme | reannealing T |
| --- | --- | --- | --- | --- | --- |
| 1 | let-7a-1 | rs10739971-F | TGGACTCTGCCTTCAATC | Cac8 Ⅰ | 59℃ |
| 2 | let-7a-1 | rs10739971-R | CTATCAGACCGCCTGGAT | 795bp(A);120,675bp(G) |  |
| 3 | let7a-2 | rs629367-F | CTTGGCTTGTCCTTGTTCTT | sequencing | 59℃ |
| 4 | let-7a-2 | rs629367-R | ATCCTTGTACCCATGCTGTA |  |  |
| 5 | let-7a-2 | rs1143770-F | TATTGTTGGGTGGATGTT | Hinc Ⅱ | 58℃ |
| 6 | let-7a-2 | rs1143770-R | GAGCAAATACTTGGGACT | 330bp(T);220,110bp（C） |  |
| 7 | let-7f-2 | rs17276588-F | ACAGAGCCAGTCGGAGAAGA | Faq Ⅰ | 58℃ |
| 8 | let-7f-2 | rs17276588-R | ATGATCGTGCCCATCCCTAA | 544bp(A);296,249bp(G) |  |

3. Sequenom MassARRAY(He et al., 2013)

Genomic DNA was diluted to working concentrations of 50 ng μL^–1^ for miRNA polymorphism genotypes. The genotyping assay was performed by CapitalBio (Beijing, China) using the Sequenom MassARRAY platform (Sequenom, San Diego, CA, USA). 10% samples were repeatedly genotyped and the concordance rate was 100%, demonstrating that the genotyping was correct.

*The detection of serum H.pylori-IgG titer*

Serum level of anti-H.pylori-IgG titer was detected by commercial enzyme-linked immunosorbent assay (ELISA) kit according to standard protocol (Biohit Co, Ltd., Helsinki, Finland) (Gong et al., 2010). If the titer was 34EIU (the cut-off value given by the protocol) or above it, it would be determined to be positive. Briefly, first, dilute the serum samples 1 to 200 (5μL+995μL) with the diluent buffer, and mix well. Second, mix and add 100μL the blank solution, the calibrators, the controls and diluted samples into the wells. Cover the plate with the incubation cover, and incubate for 30 mins at 37℃. Third, wash the wells five times with 350μL of the diluted (1 to 100 washing buffer and gently tap the plate several times on a filter paper. Then, add 100μL mixed conjugate solution into the wells, incubate for 30 mins at 37℃. After incubation, wash again and add 100μL of the mixed substrate solution into the well and incubate for 30min at room temperature (20-25℃) at a dark environment. At last, add 100μL of the mixed stop solution and measure the absorbance at 450nm within 30 mins.

*RNA Extraction and Real-time PCR reaction for miRNA expression in vivo*

1. RNA Extraction

200 μl of serum was mixed with 400 μl of phenol, 400 μl of chloroform, and 800 μl of RNase-free water. The sample was resuspended and kept at room temperature for 20 mins. The mixture was centrifuged at 12,000×g for 15 min, and the upper aqueous layer (800μl) was collected. Subsequently, a 1/10-volume of 3 mol/L sodium acetate and a two-fold volume of isopropyl alcohol were added. The total RNA was precipitated after incubation at -20^o^C overnight. The RNA pellet was collected by centrifugation at 12,000×g for 20 min, washed once with 1ml of 75% ethanol, dried for 5 min at room temperature, and then dissolved in 20 μl of 65℃ RNase-free water[4].

50ng of tissue was cut into pieces and mixed well with 1ml of Trizol (Life Technology, ), added 200μl of chloroform and vortex well for 30s. Then, the mixture was centrifuged at 12,000×g for 5 min at room temperature. The upper aqueous layer was collected and mixed with 700μl of isopropyl alcohol, kept at room temperature for 5 mins. And then, centrifuge at 12,000×g for 5 min at room temperature, the upper aqueous layer was decanted. Add 700μl of 70% DEPC RNase-free water twice, and centrifuge at 12,000×g for 2 min at room temperature. Then, the tube was inverted on clean absorbent paper for 5mins and the total RNA was dissolved in 20 μl of 65℃ RNase-free water.

2.Quantitative RT-PCR analysis of mRNA expression

1.5ug of isolated total RNA was converted into cDNA using One Step Prime Script miRNA cDNA (Perfect Real Time) (TAKARA Biotechnology Co., Ltd, Dalian, China). Briefly, 2×miRNA Reaction Buffer Mix 10μl,0.1%BSA 2μl,miRNAPrimeScript RT Enzyme Mix 2μl,Total RNA 6μl were mixed and vortex well for 30s. The reverse-transcribed reaction condition was 37℃ 60min, 85℃ 5sec.

The miRNA levels were examined using miRcute miRNA qPCR detection kit (SYBR) (TIANGEN Biotech Co., Ltd, Beijing, China) in an Eppendorf Mastercycler Gradient System (Eppendorf AG, Hamburg, Germany) according to the manufacture’s protocol. Briefly, Rnase free ddH_2_O 7.2ul, SBYR 10ul, prime 1 and 2 0.4ul, respectively, cDNA 2ul were mixed and vortex well for 30s. The Real-time PCR reaction condition was 94℃ 1min, 45 cycles of 94℃ 20s and 60℃ 34s. Melting curve analysis was performed to exclude the presence of non-specific products and primer-dimers. Each reaction was performed in duplicates and no-template controls were included in each experiment. To calculate the expression levels of the target miRNA (let-7a), the synthetic miRNA oligonucleotides (commercial miR-16, TaKaRa Inc., Dalian, China) at known concentrations were also reverse-transcribed and amplified. The concentration of each miRNA was then calculated according to the standard curve. For each assay, the resulting C_T_ values were plotted versus the log10 of the amount of miR-16(Liu et al., 2011).

*Transient transfection and Real-time PCR reaction for miRNA expression in vitro*

Plasmid Preparation: The sequenced successfully E. coli species 5ul was inoculated in 5ml LB medium at 37℃ for shaking 225rpm×16hour.

Plasmid Extracted: The plasmid was extracted using PureYield Plasmid Miniprep System (Promega, USA) by manual operation, briefly as follows: add 600μl of bacterial culture to a 1.5ml microcentrifuge tube and add 100μl of Cell Lysis Buffer with it. Add 350μl of cold Neutralization Solution and mix well. Centrifuge at 12,000×g for 3 min at room temperature and transfer the supernatant to a PureYield Minicolumn without disturbing the cell debris pellet. Place the minicolumn into a Collection Tube, and centrifuge at maximum speed in a microcentrifuge for 15 seconds. Discard the flowthrough, and place the minicolumn into the same Collection Tube. Add 200μl of Endotoxin Removal Wash (ERB) to the minicolumn. Centrifuge at 12,000×g for 15 seconds. Add 400μl of Column Wash Solution (CWC) to the minicolumn. Centrifuge at 12,000×g for 30 seconds. Transfer the minicolumn to a clean 1.5ml microcentrifuge tube, and then add 30μl of Elution Buffer to the minicolumn matrix. Let stand for 1 min at room temperature and centrifuge for 15 seconds to elute the plasmid DNA.
 Cell culture: SGC-7901 cell was cultured using 1640 cultivation and AGS was cultured using F12 cultivation with 10% serum and two kinds of antibody containing (Penicillin and [streptomycin](app:ds:streptomycin)). The 6-well plate was used to culture cells at the first day. At the second day, the transfection was used liposome lipofectine 2000 (Life Technology, USA) when cells were grown to 80% confluent. The expression plasmid pCMV-MIR-A and pCMV-MIR-C allele, were transfected into cells. Before transfection, each well of culture cells was cultured hunger without serum and antibiotics for 2hour. Plasmid 12.8 ug mixed with culture medium without serum and antibiotics 200ul for 5min; cationic liposomes 10ul mixed with culture medium without serum and antibiotics 200ul for 5min. They two were mixed for 20mins. Each sample was repeated three times. The experiment was repeated three times. After transfected cells cultured 72hour, according to the tissue RNA extraction methods (as described above), total RNA was extracted, reverse-transcribed reaction and the detection of Real-time PCR reaction were performed as described above for the let-7a expression levels.

**Supplementary Methods Reference**

Gong, Y.H., Sun, L.P., Jin, S.G. & Yuan, Y. (2010). Comparative study of serology and histology based detection of Helicobacter pylori infections: a large population-based study of 7,241 subjects from China. *Eur J Clin Microbiol Infect Dis*, **29,** 907-11.

He, C., Tu, H., Sun, L., Xu, Q., Li, P., Gong, Y., Dong, N. & Yuan, Y. (2013). Helicobacter pylori-related host gene polymorphisms associated with susceptibility of gastric carcinogenesis: a two-stage case-control study in Chinese. *Carcinogenesis*, **34,** 1450-7.

Liu, R., Zhang, C., Hu, Z., Li, G., Wang, C., Yang, C., Huang, D., Chen, X., Zhang, H., Zhuang, R., Deng, T., Liu, H., Yin, J., Wang, S., Zen, K., Ba, Y. & Zhang, C.Y. (2011). A five-microRNA signature identified from genome-wide serum microRNA expression profiling serves as a fingerprint for gastric cancer diagnosis. *Eur J Cancer*, **47,** 784-91.

Xu, Q., Yuan, Y., Sun, L.P., Gong, Y.H., Xu, Y., Yu, X.W., Dong, N.N., Lin, G.D., Smith, P.N. & Li, R.W. (2009). Risk of gastric cancer is associated with the MUC1 568 A/G polymorphism. *Int J Oncol*, **35,** 1313-20.
